# Treatment of Seabies

**Published:** 1866-06

**Authors:** D. L. Phares

**Affiliations:** Newtonia, Miss.


*Treatment of Scabies.*—Under this caption, on page 47 of the March number, is an extract from Dr. Nicholls, describing the plan of treatment introduced some ten or more years ago, by Dr. Bourguignose, of Belgium. Dr. B. claims that it will cure itch in *half an hour*, which is doubtless true of many cases. The application of the fluid should be preceded by soap and warm water bath, with constant careful friction of the entire skin for half an hour or more. Then the preparation should be rubbed into the skin for half an hour. As it evaporates, the skin is left coated with sulphur, the epizoa are killed, and the patient is cured.

I have sometimes prescribed this treatment, but oftener another similar to that of Dr. Hebra, of Vienna; both with very satisfactory results. I have, however, used most frequently the method of Helmerich, as improved by M. Hardy, effecting a radical cure of the worst cases in *two hours*. It is as follows: Rub with soft soap for half an hour the entire surface; follow with a warm bath for an hour, rubbing well all the while to break up the burrows; then rub in the ointment for half an hour, and the treatment is completed. The ointment is made by mixing together two parts of sulphur, one part of carbonate of potash and eight parts of lard. I have sometimes used the bicarbonate of potash instead of the carbonate with the same result.

D. L. PHARES, M. D.

Newtonia, Miss., May 1, 1866.

